# Low Dosage of Chitosan Supplementation Improves Intestinal Permeability and Impairs Barrier Function in Mice

**DOI:** 10.1155/2016/4847296

**Published:** 2016-08-17

**Authors:** Guiping Guan, Hongbing Wang, Hanhui Peng, Guanya Li

**Affiliations:** ^1^College of Bioscience and Biotechnology, Hunan Agricultural University, Changsha, Hunan 410128, China; ^2^Hunan Institute of Animal and Veterinary Science, Changsha, Changsha 410131, China

## Abstract

The purpose of this study was to explore relationships between low dose dietary supplementation with chitosan (COS) and body weight, feed intake, intestinal barrier function, and permeability in mice. Twenty mice were randomly assigned to receive an unadulterated control diet (control group) or a dietary supplementation with 30 mg/kg dose of chitosan (COS group) for two weeks. Whilst no significant differences were found between the conditions for body weight or food and water intake, mice in the COS group had an increased serum D-lactate content (*P* < 0.05) and a decreased jejunal diamine oxidase (DAO) activity (*P* < 0.05). Furthermore, mice in COS group displayed a reduced expression of occludin and ZO-1 (*P* < 0.05) and a reduced expression of occludin in the ileum (*P* < 0.05). The conclusion drawn from these findings showed that although 30 mg/kg COS-supplemented diet had no effect on body weight or feed intake in mice, this dosage may compromise intestinal barrier function and permeability. This research will contribute to the guidance on COS supplements.

## 1. Introduction

Chitosan (COS) is an alkaline glucosamine polymer derived from hydrolysed chitosan [1]. In common with other plant polysaccharides, chitosan molecules have a number of biological activities, such as antibacterial property, immune effect, and disease prevention [2].

Typically, peptides and proteins are not well absorbed through the intestinal tract; chitosan has been demonstrated to promote intestinal absorption of macromolecules significantly. The mechanisms behind the enhanced absorption are thought to relate to mucoadhesion and relaxing intercellular tight junctions [3]. Mucoadhesion describes the adhesion between two materials, where one is a mucosal surface. The mucus membrane bears negatively charged sialic acid groups with interacting with chitosan's positively charged amino groups [4].

Chitosan is an attractive additive for animal feed [5] because of its inherent antimicrobial properties [5], but it is restrictively used due to high viscosity and solubility [6]. Studies of the effect of chitosan on piglet growth attributed the influence on immune ability, morphology of the intestine, and microflora function [7, 8]. The results were variable and the dosages used in the studies were large (100 mg/kg–5 g/kg). Our earlier study used low dose chitosan (30 mg/kg) to supplement the diet piglets, which resulted in small intestine oxidative and immune stress responses and a reduction in intestinal barrier efficiency [9]. For this study, we predicted that a low dose chitosan supplement would disturb the intestinal permeability and function by the determination of genes expression of barrier function and proteins in mice.

## 2. Materials and Methods

### 2.1. Experiment Design

This study was conducted in accordance with the guidelines of the Laboratory Animal Ethical Commission of the Chinese Academy of Sciences. Six-week-old ICR mice were bought from SLAC Laboratory Animal Central (Changsha, Hunan, China). The mice were kept in clean animal colonies (temperature, 25°C, relative humidity, 53%; 12-h dark/12-h light); the mice had* ad lib *access to water and a typical rodent diet [10]. After three days, mice were randomly allocated to COS group (*n* = 10) or control group (*n* = 10). For two weeks, control group mice received basal diet [10], whilst the diet in the experimental group was supplemented with 30 mg/kg chitosan. Chitosan was obtained from the Dalian Chemical and Physical Institute (Chinese Academy of Sciences, Dalian, China); molecules were composed of 5 oligomers with an average molecular weight of 1,000 to 2,000 Da, a minimum sugar content of 85%, and 99% water soluble. After two weeks, the mice were sacrificed and the jejunum was recovered. Samples were frozen and kept at –80°C until required.

### 2.2. Analysis of Intestinal Permeability

The serum level of D-lactate was assessed using a commercial kit from Sino-German Beijing Leadman Biotech Ltd., Beijing, China, and a Beckman CX4 chemistry analyser (Beckman Coulter, CA, USA). Serum diamine oxidase activity experiment was established by assay kits, which were used as per the manufacturer's instructions (Nanjing Jiancheng Bioengineering Institute, Nanjing, Jiangsu, China).

### 2.3. RT-PCR of Tight Junction Genes

Complete RNA was recovered from ground jejunum using TRIzol reagent (Invitrogen, USA) and treated with DNase I (Invitrogen, USA) as per the instructions provided by the manufacturer. cDNA synthesis was carried out using oligo (dT) 20 and Superscript II reverse transcriptase (Invitrogen, USA). Primers were selected based upon previous research [10]. *β*-actin was chosen as a reference gene.

### 2.4. Immunoblotting of Tight Junction Proteins

Following the instructions provided with the total protein extraction kit (KGP200, Keygen Biotech, Nanjing, Jiangsu, China), the total protein was isolated. Equal quantities of intestinal mucosa proteins were isolated using a polyacrylamide gel before being moved onto a polyvinylidene difluoride (PVDF) membrane (Millipore, Bedford, MA, USA). These were then incubated with primary antibodies (goat polyclonal claudin-1 antibody, rabbit polyclonal ZO-1 antibody, and *β*-actin rabbit antibody) (Santa Cruz Biotechnology, CA, USA) for 12 h at 4°C. PVDF membranes were subsequently incubated with the secondary antibodies (goat anti-rabbit IgG-HRP and rabbit anti-goat IgG-HRP) (Santa Cruz Biotechnology, CA, USA) for 120 min at 25°C. Western blots were visualised using an enhanced chemiluminescence detection kit (Amersham, Arlington Heights, IL, USA) and photographed with Alpha Imager 2200 software (Alpha Innotech Corporation, CA, USA). *β*-actin reference proteins were equally distributed between the groups. The value of protein expression was determined as the densitometry ratio of tight junction proteins and *β*-actin.

### 2.5. Statistical Analyses

The statistical data analyses were carried out using SPSS 22.0 (Chicago, IL. USA). Student's *t*-test was employed to determine differences between the groups and significance was determined at *P* < 0.05. Data was presented as the means ± the standard error of the mean (SEM).

## 3. Results

### 3.1. Low Dosage Chitosan Has No Effect on Body Weight and Food Intake

**Figure 1:**
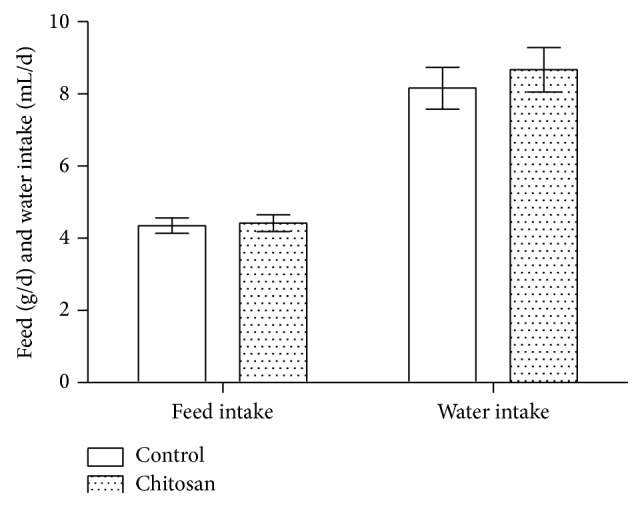
In the mouse model, 30 mg/kg chitosan supplement has no effect on food and water intake. Average intakes for each group are indicated. For two weeks, control group (*n* = 10) mice received a basal diet and the COS group of mice (*n* = 10) received 30 mg/kg chitosan supplemented diet.

**Figure 2:**
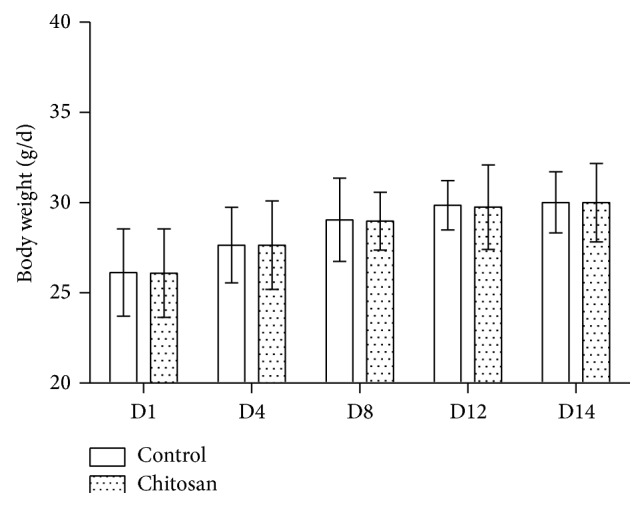
Body weight of mice for the control group and COS group averaged over the 14-day period. Control group (*n* = 10) mice received a basal diet and the COS group of mice (*n* = 10) received 30 mg/kg chitosan supplemented diet for two weeks.

The effect of a COS-supplemented diet on mice body weight, food, and water intake was determined. After two weeks, no difference in food and water intake (Figure 1) and body weight (Figure 2) was detected between the groups.

### 3.2. Low Dosage Chitosan Increases Intestinal Permeability

**Figure 3:**
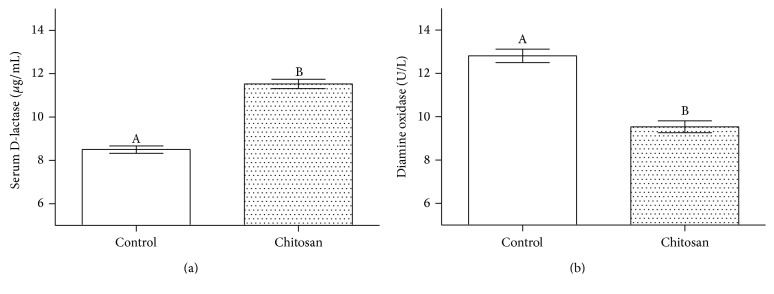
The effects of chitosan dietary supplement on (a) serum D-lactase and (b) jejunum mucosal diamine oxidase. A and B: different letters indicate a statistical difference between the control group and COS group.

To appraise the integrity of the intestine, the biomarkers of serum D-lactic acid level and jejunal diamine oxidase were measured.  Figure 3 indicates that serum D-lactate in the COS group was greater than it was in the control group (*P* < 0.05) and jejunal diamine oxidase activity in the COS group was lower than that in the control group (*P* < 0.05).

### 3.3. Low Dosage Chitosan Decreases the Expression of Tight Junction Genes and Proteins

**Figure 4:**
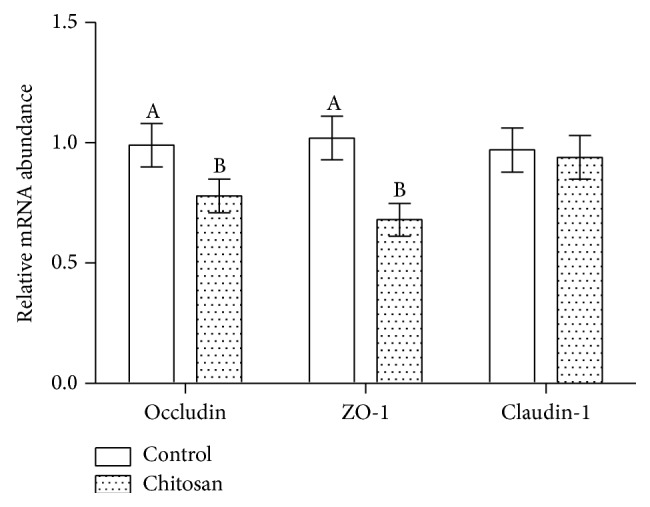
Effect of dietary chitosan on mRNA levels of occludin, ZO-1, and claudin-1 in the control (*n* = 10) and COS groups (*n* = 10). A and B: different letters indicate a statistical difference between the control and COS group.

**Figure 5:**
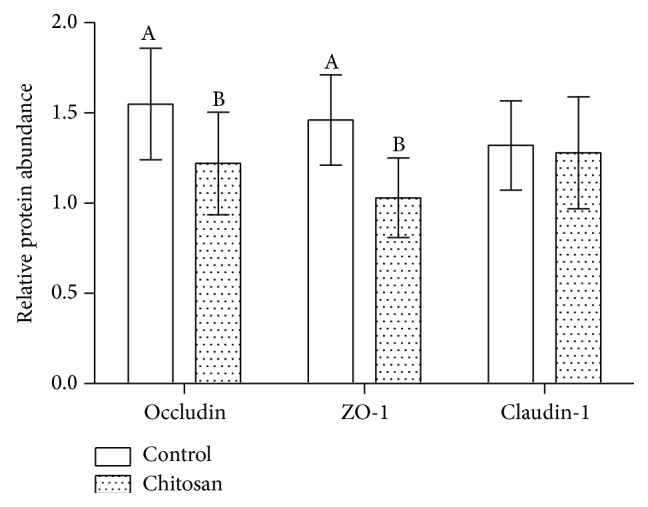
The relative abundance of tight junction proteins in the control and COS groups. A and B: different letters indicate a statistical difference between the control and COS groups.

ZO-1, claudin-1, and occludin are intestinal tight junction proteins, essential to maintain tight junction stability, and barrier function. In this study, the jejunal mucosa levels of ZO-1, claudin-1, and occludin mRNA were evaluated. The mRNA expression levels for all intestine segments are shown in Figure 4. Compared to the control group, jejunal expression of ZO-1 and occludin in COS group was significantly lower in the experimental group (*P* < 0.05). The same trend was observed in western blots via estimating the relevant protein levels (Figure 5). Compared to mice fed with control diet, the protein levels in the jejunum of ZO-1 and occludin were significantly reduced in the COS group (*P* < 0.05).

## 4. Discussion

Chitosan is the second most abundant polymer in nature [11] (cellulose being the first), which is also one of the few positively charged alkaline polysaccharides. Chitosan as a dietary supplement has been explored for its potential as an antimicrobial growth promoter and has been shown to promote growth in broiler chickens [12, 13] and pigs [14]. This could be ascribed to higher feed intake, serum growth hormone, and IGF-1 concentrations [15]. In this study, we found that a low dosage COS-supplement diet did not have any impact upon food and water intake or body weight in mice. This echoes the finding of our earlier study that no effect on the average feed and body weight was found via using the same 30 mg/kg dose of chitosan to supplement piglet diets [9]. Huang et al. also reported that no difference in growth performance was found in a broiler chicken study that used a high chitosan dose (150 mg/kg) [12]. Differences between the dosage, molecular weight, purity, or solubility of the chitosan used in this experiment and others may account for the contrary results [16].

To determine the effect of chitosan on intestinal integrity, we evaluated the concentration of serum D-lactate and jejunal diamine oxidase in accordance with these biomarkers which are identified as useful to determine gut integrity [17]. Reduced intestinal diamine oxidase activity and elevated serum D-lactate levels corresponded with cell and tissue injury [18]. We used the same biomarkers to evaluate the permeability of the intestine. Our results indicated an increase in the serum level of D-lactate and activity of diamine oxidase in response to 30 mg/kg dose of chitosan; this suggests that intestinal integrity is compromised.

The epithelium of the intestine forms a selectively permeable barrier that is key in preventing pathogenic invasion. Tight junctions are central to the barrier's functionality and are responsible for its integrity [19]. Tight junction proteins encompass a number of integral membrane proteins that are bound to cytoplasmic plaque proteins.

Occludin, claudin-1, and ZO-1 are tight junction proteins that vary in their molecular structures and function; they are collectively important to maintain the tight junction's structure and function. Occludin and claudin-1 are transmembrane proteins that regulate the tight junction's function and integrity [20]. It has been demonstrated by Shen et al. that the cytoskeleton regulates permeability of the leak pathway via mechanism that includes occludin and ZO-1 [19]. On the other hand, Rosenthal et al. demonstrated that paracellular permeability could be modified by chitosan, independent of changes to tight junction proteins [21]. In this study, we detected changes to the mRNA expression of ZO-1 and occludin in the group of mice that had been fed a COS-supplemented diet. In the COS group, jejunal mRNA expression of occludin was lower and expression of ZO-1 was reduced. This observation is consistent with the raised concentration of serum D-lactate and decreased level of jejunal diamine oxidase. However, mRNA expression of claudin-1 was unaffected. These findings imply that in mice the integrity of the intestinal barrier is compromised by low doses of dietary chitosan supplementation.

To conclude, in the mouse model, 30 mg/kg dose of chitosan supplements did not influence growth performance but compromised intestinal barrier integrity. The findings from this research will contribute to the guidance on low dose chitosan supplements.
